# The Role of Proteomics in the Diagnosis and Treatment of Women's Cancers: Current Trends in Technology and Future Opportunities

**DOI:** 10.1155/2011/373584

**Published:** 2011-07-25

**Authors:** Eun-Kyoung Yim Breuer, Mandi M. Murph

**Affiliations:** ^1^Department of Systems Biology, University of Texas MD Anderson Cancer Center, Houston, TX 77054, USA; ^2^Department of Radiation Oncology, Loyola university of Chicago, Stritch School of Medicine, Maywood, IL 60153, USA; ^3^Department of Molecular Pharmacology and Therapeutics, Loyola university of Chicago, Stritch School of Medicine, Maywood, IL 60153, USA; ^4^Department of Pharmaceutical and Biomedical Sciences, College of Pharmacy, The University of Georgia, 240 W Green Street, Athens, GA 30602, USA

## Abstract

Technological and scientific innovations over the last decade have greatly contributed to improved diagnostics, predictive models, and prognosis among cancers affecting women. In fact, an explosion of information in these areas has almost assured future generations that outcomes in cancer will continue to improve. Herein we discuss the current status of breast, cervical, and ovarian cancers as it relates to screening, disease diagnosis, and treatment options. Among the differences in these cancers, it is striking that breast cancer has multiple predictive tests based upon tumor biomarkers and sophisticated, individualized options for prescription therapeutics while ovarian cancer lacks these tools. In addition, cervical cancer leads the way in innovative, cancer-preventative vaccines and multiple screening options to prevent disease progression. For each of these malignancies, emerging proteomic technologies based upon mass spectrometry, stable isotope labeling with amino acids, high-throughput ELISA, tissue or protein microarray techniques, and click chemistry in the pursuit of activity-based profiling can pioneer the next generation of discovery. We will discuss six of the latest techniques to understand proteomics in cancer and highlight research utilizing these techniques with the goal of improvement in the management of women's cancers.

## 1. Risk of Women's Cancers

The likelihood of developing cancer and the specific type of cancer vary tremendously during a woman's lifetime (Table 1). Among women in the USA who are between 40–79 years of age, cancer is the leading cause of mortality. This number decreases substantially for those of 80 years and older after which cancer is usurped by heart diseases for the top morbid distinction. In actuality, it is this group of 80 years and older that marginally places the ranking for heart diseases above cancer as the overall leading cause of death in the USA by approximately 53,000 people [1]. Since cancer is far more likely to occur during the prime years of a woman's life, it therefore has an enormous impact on families, future generations, business productivity, and loss to society. 

Although men and women share this grim reality together, anatomical differences between the genders naturally assign more cancer susceptibilities to women. Even with this gender factor siding against women, they are remarkably less likely than men to be diagnosed and succumb to cancer by approximately 50,000 and 29,000 each year, respectively. Some notable exceptions are lung, colon, and pancreatic cancers, which affect both groups equally [1]. Although breast cancer can occur in both men and women, women are far more likely to suffer a diagnosis or death as a result. As an interesting inverse correlation, married men with breast cancer are more likely to receive treatment and exhibit reduced mortality [2], suggesting that women play a positive supporting role for others with this malignancy. Women are additionally susceptible to cancers of the ovary, vagina, vulva, cervix, uterus, or endometrium lining the uterus. They also have a far greater prevalence for thyroid cancer (15.2 per 100,000 women) than men (5.2 per 100,000 men) [3], which is particularly troubling given that some patients report having no symptoms other than a lump on the throat. The combined figures from the aforementioned women's cancers account for 25% of the total cancer mortality among women in 2010 [1].

## 2. Breast Cancer Screening and Detection

Due to early detection, better diagnostics, and improved treatment strategies, survival rates have vastly improved for breast cancer patients within the past 30 years. In the 1970s, the 5-year survival rate associated with breast cancer was approximately 75%, and today it is over 90% [1]. Improved outcomes are further highlighted by the fact that in the USA today there are over 2.5 million women alive who have a history of breast cancer [3], a triumph to successful scientific and medical advancements. 

Breast self-exams and clinical exams for palpable masses along with mammograms are employed for the early screening of breast cancer. Most women who will be diagnosed with ductal carcinoma in situ have tumors detected through mammography [4], suggesting a prominent role for screening in the detection of this malignancy. The goal of the mammogram is to detect small masses <1 cm and calcifications [5]. Although lobular carcinoma in situ is not detectable by mammography [6], it can be assessed through core needle or excision biopsy. Lobular carcinoma in situ is unique because these tumors are not typically discovered as palpable masses and can present as mammographic calcification [7], which is why routine mammography is so important. The detection of calcification requires further analysis through additional diagnostic imaging, magnification views, sonography, 6-month followup exams, or biopsy [5]. 

Before the 2009 bombshell controversy and heated public response surrounding the USA Preventive Services Task Force initial updated recommendations for breast cancer screenings, women were recommended to have mammograms starting at age 40, unless they had a high risk family history and then the recommendation to start was at age 30. After the ensuing brouhaha, the following month the Task Force “voted unanimously to update the language of its recommendation … mammography before the age of 50 years should be an individual one and take patient context into account, including the patient's values regarding specific benefits and harms.” [8]. Further independent recommendations for the high risk group are for an additional annual screening using MRI, which is more sensitive at detecting carcinomas than both sonography and mammography, particularly in those with dense breasts. The drawbacks to MRI include the reduced detection of calcifications, the higher cost associated, and the increased time required by the instrumentation [5].

Private online risk assessments exist for those seeking no-cost answers for their health and curiosity. The National Cancer Institute hosts a website for the Breast Cancer Risk Assessment Tool (http://www.cancer.gov/bcrisktool/) which estimates the risk for invasive cancer development. This calculator only applies to women over 35 years old who have not previously had a diagnosis of breast cancer. It asks seven questions about a woman's age, first menstrual period, first childbirth, relatives, ethnicity (although it was designed with data based on white and African American females), and whether the woman has previously had a biopsy. Fair warning for users: there is no “right” answer that produces a 0% risk, and the tool is based on an average of 12.6% lifetime risk from 0 to 90 years.

## 3. Breast Cancer Biomarkers and Diagnostics

Breast cancer serves as a model for other cancers in that there are a variety of biomarker-incorporating predictive tests and therapeutic treatments to guide clinical decisions. Many cancers have no early screening, detection, or prognostic predictors available, and in that respect, breast cancer is leading the way in innovation. The Nottingham Prognostic Index is an early example of prognosis prediction among breast cancer patients based on tumor size, stage of disease, and tumor grade [9]. More sophisticated tests that are now available are summarized in Table 2 and extensively reviewed elsewhere [10]. Another recent test that was commercially launched is Mammostrat, a five-biomarker, immunohistochemical assay that measures CEACAM5, HTF9C, p53, NDRG1, and SLC7A5 [11]. The major drawbacks are that not every patient will fall exactly into the specific predictive model, meaning that a small percentage may endure unnecessary overtreatment, and also that not all of these tests have been exhaustively studied.

Clinical trials are ongoing to assess the accuracy of predictive tests in breast cancer but many of these trials will not be completed for several more years. For example, the Trial Assigning IndividuaLized Options for Treatment (Rx), or TAILORx, examines the reliability of Onco*type* DX in aiding individualized treatment options, assessing recurrence and predicting prognosis among 11,248 number of patients, but will not be concluded until 2014 [12]. For patients who are unwilling or unable to wait that long, these tests may at least provide some directive guidance for clinical conversations to seek the most appropriate individualized treatment currently available in the medical arsenal.

The breast cancer prediction tests also vary in the proteins, gene expression signatures, patient variables, clinical histology, and other biomarkers or tumor characteristics that are incorporated into the model. One essential commonality is the expression of hormonal or growth-factor-dimerizing receptors within the tumor, and this still remains the cornerstone for therapy in breast cancer. For instance, whether a tumor is positive for the oestrogen receptor (ER), progesterone receptor (PR), or the human epidermal growth factor 2 receptor (HER2, also known as the ErbB2 receptor or neu receptor) is tantamount for defining the subtype of breast cancer and assigning therapeutic intervention. Thus far, there are at least five molecularly defined subtypes, which can also categorize subtypes of breast cancers and these are luminal-A, luminal-B, Her2, basal-like, and claudin-low; it is unclear whether the controversial normal-like represents a true molecular subtype or merely a contamination of breast tumor specimens with normal mammary tissue [13–16]. 

Even with the newer classifications, hormone receptors are still the most important factor regarding treatment. This is because oestrogen is capable of driving the growth of oestrogen-dependent tumors, and inhibiting this hormonal factor is crucial to achieving a response to therapy. As discussed below, there are layered approaches along with many drugs that achieve this goal. Since protein receptors are thus the fundamental criteria upon which other decisions are based, it further highlights the critical roles proteins have within the tumor biology, assigning treatment and predicting outcomes.

## 4. Breast Cancer Prescription Therapeutics

Following a medical diagnosis of breast cancer, the presence of ER, PR, and HER2 is routinely tested and the tumor is characterized to determine what the treatment regimen will be. Breast cancer therapeutics are now so sophisticated with targeted agents that this information is absolutely critical to charting the course. In addition to the essential knowledge of whether the patient's tumor expresses ER, PR, or HER2, the tumor (or tumors) size, lymph node stage and histological grade of disease are critical and may determine whether or not the patient will receive chemotherapy. It is no longer the case that all women diagnosed with breast cancer will undergo repeated cycles of chemotherapy to treat their disease. Patients with a small breast tumor <1 cm that are diagnosed at an early-stage, and have not metastasized are likely among this group since chemotherapy would result in overtreatment and unnecessary side effects [17]. Alternatively, schedules can vary greatly as neoadjuvant, adjuvant, and dose-dense chemotherapy administrations are all possible in this disease.

For breast tumors that are hormone-sensitive (ER- or PR-positive), drugs that fall into the broad category of oestrogen inhibitors are prescribed for 5 years or even longer to reduce recurrence. Specifically, studies have shown that 2 years of adjuvant tamoxifen reduces recurrence by 14% and mortality by 10%, while 5 years further reduces these by 45% and 32%, respectively [18]. In premenopausal women, ovarian ablation can occur through treatment with luteinizing hormone-releasing agonists, radiation therapy, or surgery [19]. Although there are now other drugs available to mitigate oestrogen synthesis and signaling in both postmenopausal and premenopausal women, the sequence of first-line treatment usually starts with tamoxifen, but could also include toremifene or raloxifene. These are the antioestrogen drugs, which are also selective estrogen receptor modulators. If endocrine resistance emerges, then aromatase inhibitors like anastrozole, letrozole, exemestane, formestane or fadrozole, that prevent the synthesis of oestrogen may be used for second-line treatment and these are reviewed elsewhere [20]. Fulvestrant, which binds to the ER, causes the destruction of the receptor and is classified as a pure antagonist. Due to this unique mechanism, it can be used as either second- or third-line in the sequence of drug therapies for hormone-sensitive breast tumors. Lastly, the progestin megestrol acetate is used as third- or fourth-line therapy for advanced breast cancer because of its antiestrogenic and cytotoxic effects [19]. Taken together, the number of drugs and approaches to therapy provides many options for therapeutic regimens lasting 5 years and even 10 years for some patients.

Approximately 15–20% of breast cancer patients have HER2-positive tumors [21], meaning their tumors overexpress HER2, which dimerizes with epidermal growth factor receptors and amplifies aberrant signaling. On its own, the HER2 receptor has no independent ligand. Among these HER2-positive patients, the monoclonal antibody, trastuzumab, or the small-molecule inhibitor, lapatinib, will be prescribed to specifically target the HER2 receptor. Pertuzumab, another monoclonal antibody against the HER2 receptor, is still undergoing clinical trial testing. Thus far, in doublet combinations with trastuzumab and the chemotherapeutic agent docetaxel this combination may have enhanced efficacy with the addition of pertuzumab [22].

Other groups of patients are categorized as triple-negative breast cancer, which is ER-, PR-, and HER2-negative and accounts for approximately 15% of tumors, or advanced-stage and metastatic breast tumors. These groups of patients will require more chemotherapy than others for treatment. Triple-negative tumors are insensitive to hormonal treatments, yet investigational agents belonging to a class of poly(ADP-ribose) polymerase or PARP inhibitors, including iniparib, olaparib, and veliparib, may have some ability to achieve response [23, 24]. Most patients with advanced breast cancer that are given chemotherapy will receive the “FAC” regimen, which stands for 5-fluorouracil, doxorubicin, and cyclophosphamide. The “CMF” regimen, which stands for cyclophosphamide, methotrexate, and 5-fluorouracil, is given to patients that are unable to receive the anthracycline drug doxorubicin because of comorbid medical conditions [25]. Other drugs that could be part of a therapeutic regimen include paclitaxel, docetaxel, or epirubicin [26].

## 5. Cervical Cancer Screening and Detection

Although cervical cancer does not largely attribute to mortality in the USA [1] due to advances in early screening and detection, globally it is a major cause of death from gynecologic cancer [27]. The human papilloma virus (HPV) causes cervical cancer, and this virus is usually transmitted through contact with squamous epithelial cells which are susceptible to its infection. At least 100 or more genotypes of HPV exist, and these are divided into two types, “high risk” and “low risk,” reflecting the potential to induce invasive cancer. It is the high risk types of HPV such as HPV-16 and HPV-18 that are causally involved in developing cervical [28] and other anogenital cancers (Table 3) [29, 30]. Even though genital HPV infection is extremely common and often causes no symptoms, some of the low risk viruses like HPV-6 and HPV-11 cause recurrent respiratory papillomatosis or genital warts and might be self-diagnosed, given the location [31]. 

**Figure 1:**
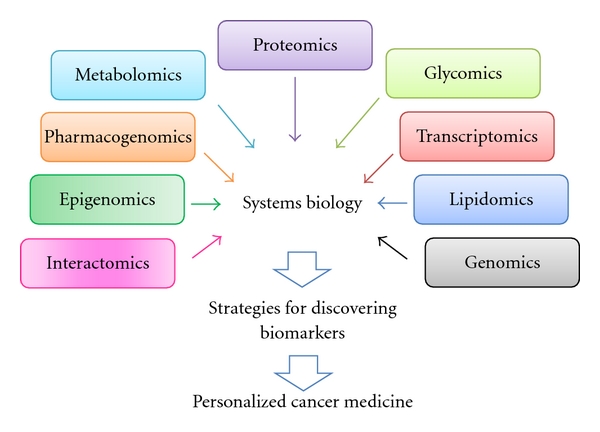
From omics-based systems biology to personalized cancer medicine.

The “Pap smear,” Pap test or Papanicolaou smear named after Dr. George Papanicolaou the inventor, is a screening technique to detect early evidence of cervical cancer. The Pap smear collects cells scrapped from the outer opening of the cervix of the uterus and then examines preps of those cells underneath a microscope to determine whether abnormalities are present in the cervix. There is also a “HPV test” that can be combined and tested simultaneously with the Pap smear to confirm the presence of the virus. Should abnormalities indeed be present, then a colposcopy is performed to evaluate the area (Figure 1). The Pap smear is the best example of a cancer screening program; however, sometimes false-positive or false-negative results do happen, leading to unnecessary followup or causing delay in the diagnosis and treatment of precancer and cancer, respectively. Therefore, alternative or complementary screening tools are needed to overcome the limitations of Pap smear and produce a better outcome.

## 6. Cervical Cancer Biomarkers and Diagnostics

There are protein biomarkers that could have utility in assessing disease risk, early detection, and prognosis in cervical cancer. For example, the HPV E6 and E7 oncogenes play an essential role in HPV-induced carcinogenesis by interfering with normal cellular events and are possible biomarkers for early detection of cervical cancer. Studies show that detection of E6/E7 mRNA expression could predict the risk of cervical cancer better than HPV DNA testing [32] and currently the commercially available mRNA-based assays (e.g., NucliSENS EasyQ HPV Test and APTIMA HPV mRNA Assay). Arbor Vita Corporation has developed a rapid diagnostic test, “AV Avantage HPV E6 test” in collaboration with PATH (the Program of Appropriate Technology in Health), a nonprofit global health agency, with FDA approval targeted in 2013. AV Avantage HPV E6 test uses a high-affinity monoclonal antibody for the detection of E6 oncoprotein from high risk HPV-16, -18, and -45 responsible for approximately 90% of cervical cancers. Small clinical pilot studies showed a potential feasibility and clinical applicability of AV Avantage HPV E6 Test [33], and further investigation is underway.

The tumor suppressor p16^INK4A^ plays an important role in regulating the cell cycle and is overexpressed in the presence of the HPV E7 oncoprotein. Several studies reported p16^INK4A^ as a useful diagnostic marker for squamous and glandular epithelial dysplasia in the uterine cervix [34, 35] and a valuable surrogate marker for high risk and malignant cervical lesions in the presence of HPV [36]. Furthermore, expression of p16^INK4A^ appears to correlate with the degree of cervical neoplasia [37, 38]. A recent study showed that a p16^INK4A^ immunocytochemical assay has better specificity than HPV testing to predict underlying high-grade dysplastic lesions [39]. Currently, clinical trials are underway to assess the diagnostic and prognostic value of p16^INK4A^ expression in atypical glandular cells and low-grade squamous intraepithelial lesions of the cervix.

Squamous cell carcinoma antigen (SCC-Ag) is a marker of squamous cell carcinomas in the cervix [40] and expressed in normal cervix epithelium with an increased expression in dysplastic lesion and cervical squamous cell carcinoma. SCC-Ag is not sensitive or specific enough for detection of early-stage cervical cancer; however, pretreatment serum SCC-Ag values serve as a strong, independent prognostic factor [41]. Several studies have concluded that SCC-Ag is useful for posttherapy surveillance monitoring of cervical cancer [42] and is currently being used in some hospitals. The persistent increase of serum SCC-Ag levels during and after radiotherapy is associated with persistent or recurrent disease [43], indicating the important implication of SCC-Ag for further diagnostic workup and clinical management [44]. 

Ki-67 is a nuclear protein that is expressed during all active phases of the cell cycle, and its expression is used to determine the cell proliferation status [30, 45]. In cervical intraepithelial neoplasia (CIN), Ki-67 expression is increased in the upper layers of cervical epithelium compared to normal cervices [30, 46, 47]. Several studies have also suggested that Ki-67 can be used as an independent prognostic marker to identify women with high risk for progression and/or recurrence of cervical squamous precancerous lesions [48]. 

The eukaryotic minichromosome maintenance (MCM) protein family consists of six essential proteins (MCM2-7), all of which are necessary for DNA replication [49, 50] and are abundantly expressed through the cell cycle [51, 52]. While MCM protein staining is limited to the basal proliferating layer and absent in differentiated cells, its expression is significantly increased in cervical glandular and squamous dysplasia [53, 54], suggesting its potential as a biomarker of cervical dysplasia. Studies have revealed a strong correlation between the number of nuclei positive for MCM2 and MCM5 at the surface of dysplastic epithelium and the severity of dysplasia [53, 55]. MCM7 has also been identified as a highly informative marker of cervical cancer. Full thickness staining for MCM7 staining was observed in high-grade cervical epithelial lesions and invasive cervical carcinoma [55, 56].

Topoisomerase II*α* (TOP2A) functions as a key enzyme in DNA replication and cell cycle progression [57]. Increased expression of TOP2A was observed in cervical disease and cancer [58], and its expression is correlated with increased risk of progression from CIN2 to CIN3 [59]. A novel reagent that detects MCM2 and TOP2A is commercially available (BD ProEx C) and is a proposed marker with the potential for detecting high-grade squamous intraepithelial lesions in cervical biopsy specimens [60]. Interestingly, the performance of BD ProEx C as a complementary surrogate marker to p16^INK4A^ and Ki-67 showed improved diagnostic accuracy [60], suggesting its potential clinical use. Currently, BD ProEx C and other molecular markers are being investigated to improve cervical cancer diagnosis.

## 7. Cervical Cancer Prescription Therapeutics

Currently, there are two HPV vaccines on the market, Gardasil and Cervarix. The approval of these novel therapeutics championed the first bona-fide cancer prevention drugs approved by the FDA. Both vaccines protect against the acquisition and infection of HPV-16 and -18, the types of viruses responsible for approximately 70% of cervical and other genital cancers [61]. Additionally, Gardasil also protects against HPV-6 and -11 which cause approximately 90% of genital warts and potentially prevents precursors to penile, vulvar, vaginal, and anal cancers [61]. The major drawback of these vaccines is that they cover the most common types of HPV, but not all 100-plus genotypes. Thus, vaccinated women would still need to have regular screening tests routinely performed to ensure they were not at risk for cervical cancer. In other words, the vaccines do offer a level of protection, but are not enough to eliminate all risks of HPV infection. It is unclear whether infrequently appearing HPV types will become more prominent among the human population as a long-term result of these vaccines. Furthermore, without 100% compliance from the population in supporting these somewhat controversial vaccines, it is unclear how effective they will be on reducing incidence of cervical cancer, the costs of screening and detection, and anxiety surrounding positive- or false-positive Pap smear results.

Cervical cancer is staged by the Federation of International Gynecology and Obstetrics (FIGO) system, which begins at 0 and ends at IV. In advanced stages of disease, like most other types of cancer, chemotherapy will be administered to the patient to control the growth and spread of tumors. If early abnormalities of the cervix are not removed, these abnormalities or precancerous lesions could develop into carcinoma *in situ*, low-grade or high-grade CIN and then into cancer. For cervical cancer, stage I–III disease will likely require combination regimens with cisplatin and another agent, either 5-fluorouracil, topotecan, paclitaxel, vinorelbine, or irinotecan [26]. Radiation therapy may also be part of the treatment plan. For patients with stage IV disease, a barrage of chemotherapy available in the oncology arsenal may be used.

## 8. Ovarian Cancer Screening and Detection

An unfortunately and sometimes heart-wrenching clinical reality is that no early screening or early diagnostic test exists to indicate a woman has ovarian cancer. The Papanicolaou test or “pap smear” will not detect ovarian cancer, and even routine well woman exams have failed to detect malignancy, much to the newly diagnosed patient's distress and frustration. One reason outcomes are generally poor is that the majority of patients present with late-stage disease due to very general symptoms that may persist for years and could be attributed to other factors. For example, the most frequent symptoms reported in the clinic include abdominal bloating, pain or swelling, dyspepsia, urinary frequency and significant or unexpected weight change. Other symptoms reported by patients include painful intercourse, a change in bowel habits, backaches, and fatigue. Thus, these symptoms might not elicit significant attention from the patient until the disease has spread and developed into a later stage, causing the symptoms to be unbearable. This is the rationale behind public education and awareness programs that have adopted whisper campaigns to encourage women to notice the combination or subtle changes among normal bodily functions. Alternatively, a practitioner could misdiagnose the generalizable symptoms as more common maladies, such as irritable bowel syndrome.

Most tumor staging systems use the convention that spreading from the primary tumor describes a metastatic, stage IV disease. This is not necessarily the system that has evolved for ovarian cancer, which uses the FIGO system. Ovarian cancer staging occurs during the surgery and is based on operative conclusions observed, whereby malignant cells can be found in peritoneal washings as early as stage Ic and metastases to the uterus is categorized as stage IIa [62]. In addition, the fact that malignant cells shed and passively move through the peritoneal cavity so early in malignancy generates significant challenges for treatment.

## 9. Ovarian Cancer Biomarkers and Diagnostics

Corresponding with the problem of unavailable diagnostics for this malignancy, there is also a lack of biomarkers due to the specificity requirement of this rare disease. Many worthy ideas have not been able to achieve sufficient levels of sensitivity and specificity in ovarian cancer, leaving women without any reliable means of early detection. In addition, there have been many problematic ovarian cancer biomarkers that did not live up to the ground-breaking status original perceived, which could not be validated upon subsequent examination [63]. There are, however, plenty of innovative biomarker ideas that have not yet been rigorously examined in clinical trials [64, 65] to determine utility and at least one older example of a classical discovery which is being used in a new way.

This classical discovery surrounds CA-125, which is also known as cancer or carbohydrate antigen 125, and may be elevated when malignant disease is present. CA-125 is actually mucin 16, a carbohydrate glycoprotein that is expressed and shed by the cells, but lacks specificity and sensitivity to be used as a screening tool for ovarian cancer. The originally discovery of CA-125 occurred when investigators developed and tested OC125, a clone that produces an IgG1 murine monoclonal immunoglobulin that reacted with 6 of 6 epithelial ovarian cancer cell lines and patient tissue [66]. At the time, investigators were searching for an immunotherapy and/or a unique tumor-associated antigen on the cell surface for immunodetection; this discovery was the 125th attempt. 

Although the novel discovery was found to be useless as a treatment, CA-125 showed some ability as a biomarker. The majority of ovarian cancers shed CA-125, which is the basis for this association. In its currently use, patients previously diagnosed with ovarian cancer will have blood levels measured for CA-125 to indicate disease recurrence, monitor disease progression, response to treatment, or predict prognosis after treatment. In these situations it is considered a generally reliable tool for clinicians, even though small percentages of women with ovarian cancer will not have significantly elevated levels. Prior to surgical debulking, ovarian cancer patients might have CA-125 that exceeds 2000 units (some patients even exceed 10,000 units) while most normal healthy women have levels of CA-125 <35 units in circulation. There are conditions unrelated to ovarian cancers that cause a rise in CA-125, and this limits the utility of the measurement. For example, normal ovarian, pancreatic, and breast cells along with tissues lining the abdomen and chest make and release low levels of CA-125. In addition, diverticulitis, pelvic inflammatory disease and pancreatitis are all examples of abdominal conditions that increase CA-125 and are noncancerous.

A clinical trial recently assessed whether starting second-line chemotherapy based on CA-125 level elevation, not the appearance of symptoms, would affect outcomes by theoretically beginning treatment earlier with recurrent disease. Surprisingly, the results failed to demonstrate an increase in survival in the application of this idea [67]. Some critics charged that not sorting patients based on computed tomography scans could have affected the results and that guidelines for monitoring patients' CA-125 levels should not be changed based on this study [68]. Controversies surrounding the use of nonperfect biomarkers are not only applicable to CA-125; the PSA test has encountered similar criticism [69].

What is the best utility of CA-125, and can it be used in other ways to help provide clinical guidance for the early detection of ovarian cancer? A prospective study performed at the University of Texas MD Anderson Cancer Center sought to address this question by considering the change in CA-125 over time. The clinical trial stratified women into three groups of low, mid, and high risk, based upon a baseline CA-125 reading, the current measurement, and the woman's age. Using the results from the calculated risk, women in the high risk group received transvaginal sonography and it was determined that five women indeed had early-stage ovarian cancer [70, 71]. This suggests that CA-125 may have a place in diagnosis when coupled with other diagnostic technologies and/or biomarker tests. If such techniques like transvaginal ultrasound coupled with biomarker detection can enhance screening or diagnostics of ovarian cancer, then ongoing clinical trials may elucidate this possibility. Recently it was suggested that transvaginal ultrasound screening for women at high risk of endometrial cancer could have a role in patient management [72]. In this case, the screening is designed to measure increasing endometrial thickness and other abnormalities. In a large case-controlled study that included more than 40,000 women, transvaginal ultrasound demonstrated good sensitivity in postmenopausal women, although the report of results stopped short of recommending population screening [72].

In 2009 the FDA approved the first blood serum test, OVA1, designed to assist determining whether a suspicious pelvic mass is malignant ovarian cancer prior to a planned surgery. The test is not intended for screening and cannot be used alone. It is approved in combination with standard surgical evaluations, which seems to reduce its usefulness; however, the utility of OVA1 occurs in cases where other clinical tests do not indicate malignancy when it is present. OVA1 uses five immunoassays to derive an independent score (0–10, indicating no risk to highest risk) based on proprietary software called OvaCalc, and this score suggests the likely risk of malignancy, but is not considered a diagnosis of ovarian cancer. The five proteins that the assay measures include apolipoprotein A-1, beta2-microglobulin, transferrin, transthyretin, and CA-125 (http://www.ova-1.com/) [73]. 

In 2010, an FDA-approved test for human epididymis protein 4 (HE4), a secreted protease, became available for monitoring recurrence or progression of ovarian cancer. Previously, HE4 was touted as a biomarker for ovarian cancer; furthermore, measurements of HE4 successfully detected malignancy with 67% sensitivity and 96% specificity [74]. To enhance the monitoring capability of HE4, other assays that measure mesothelin and/or CA-125 are being experimentally tested and clinically evaluated to determine whether a combinatorial test has clinical utility above HE4 alone, particularly in suspect patients with a pelvic mass [75, 76]. When HE4 was combined with CA-125 and two other biomarkers, the four-panel set was able to diagnose late-stage ovarian cancer with a sensitivity of 93% and specificity of 98% [65], suggesting the combination is superior to any one biomarker alone.

## 10. Ovarian Cancer Prescription Therapeutics

Although adjuvant chemotherapeutics for ovarian cancer are capable of reducing disease to undetectable levels in combination with surgical debulking, the therapeutic modalities are not nearly as sophisticated as those of breast cancer. In fact, the most impactful breakthrough for this disease occurred in the late 1970s when cisplatin was introduced into clinical trials and received rapid FDA approval for this indication. At that time, several clinical studies using cisplatin demonstrated its ability to increase response rates, relieve symptoms from ovarian tumors, and improve survival [77–79]. The next major breakthrough occurred in the early 1990s when paclitaxel or taxol, a product derived from bark on the Pacific yew tree, was FDA approved after clinical studies suggesting activity against this malignancy [80, 81]. Due to the favorable safety profile and efficacy of Taxol after its initial discovery period, unprecedented demand caused drug shortages and the National Cancer Institute established plans to increase the drug's supply through partnerships and commercialization [82, 83].

The culmination of these discoveries lead to the establishment of first- and second-line chemotherapeutic regimens for ovarian cancer used today, the former which is six courses of a platinum- and taxane-based combination [17]. For women who are diagnosed with stage IIa or greater stages of ovarian cancer, all of the following are possible in addition to surgical debulking: total abdominal hysterectomy, bilateral salpingo-oophorectomy, omentectomy, pelvic irradiation, and systemic chemotherapy. The recommendation for systemic chemotherapy is in contrast to the minority of women who are diagnosed with stage Ia or Ib with grade 1 and 2 lesions where chemotherapy is not recommended [62]. 

Second-line chemotherapy largely depends on how the patient responded to first-line treatment. This is due to the fact that the first-line chemotherapy regimen may be used again when the patient reappears in the clinic with the same malignancy. For example, the categories include platinum-sensitive, partially platinum-sensitive, platinum-resistant, and platinum-refractory disease [17] which will guide whether cisplatin or carboplatin is administered. Since the majority (approximately 75%) of ovarian cancer patients will relapse, some with refractory disease, the establishment of appropriate second-line therapy is critical. If a patient is grouped into the platinum-refractory disease category, then other agents like liposomal doxorubicin, topotecan, gemcitabine, etoposide, or single-agent paclitaxel may be administered [26]. Clinical trials are underway assessing the response rate when administering bevacizumab or PARP inhibitors in this malignancy.

## 11. New and Emerging Proteomics Techniques in Cancer Research including Prognostic Techniques

In the postgenomic era, clinical proteomics strategy for deciphering and characterizing the diseased proteome network is clearly becoming a next major challenge to better understand the aberrant changes that cause cancer, predict a patient's risk of developing certain types of cancer, predict outcomes, and guide treatment decisions. The recent advancement in clinical proteomics technologies shows great promise for improvement in all of those areas, eventually leading to better patient outcomes; however, studies applying proteomics technologies to clinical applications have suggested there are big challenges to overcome. Therefore, researchers are increasingly interested in using a combination of proteomics and other “OMICS” technologies that have showed feasibility in the clinical setting, but further evaluation and validation is necessary (Figure 1). 

As more is discovered about DNA, RNA, and small RNAs, the latter which appears to be a constantly growing field, it is realized that the end point is a change in protein. Protein changes caused by gain- or loss-of-function mechanisms via, for example, protein-protein interaction or posttranslational modification may contribute to the etiology of cancer. Due to the complexity of proteins as well as the dynamic nature of the proteome in human body, traditional proteomic approaches have been a difficult task. For these reasons, proteomics technology is being extensively explored and has now become an emerging field in cancer research. It is the final frontier of “OMICS” research, the missing puzzle piece. Proteomics has led and will continue to lead us all toward the future of personalized cancer management. In this section below, we will discuss emerging new proteomics strategies and implications for cancer research.

## 12. Protein Analysis Using Mass Spectrometry

Mass spectrometry offers a number of advantages for the biochemical analysis of lipids [84, 85], nucleic acid fragments, [86] and proteins, thus it has become a powerful and indispensable tool for proteomic studies [87–89]. This innovative and pioneering technique has taken protein analysis to the next level, beyond the days when laboratories relied on one- and two-dimensional gel electrophoresis capable of resolving a few hundred proteins, which would then require sequencing. Mass spectrometry is capable of resolving thousands of proteins and thus allows scientists to quantitatively probe the proteome, oncoproteome, or secretome on an in-depth level that was previously impossible. On another note, mass-spectrometry has revolutionized forensic science through the analysis of hair fibers, gunshot residues, and chemical detection used in drug testing, arson investigations, and explosives. Despite the recent technological advances of mass-spectrometry, great challenges are still presented in establishing high-resolution, accurate mass-spectrometry methods for quantitative bioanalysis. Over the past several years, many novel mass-spectrometry-based quantitative proteomic methods have been developed for quantitative analysis of relative differential changes in protein abundance.

## 13. Stable Isotope Labeling with Amino Acids in Cell Culture (SILAC)/Super-SILAC

Among the many formats of quantitative proteomics methods, stable isotope labeling with amino-acids in cell culture (SILAC) has become a powerful and versatile tool [90]. The SILAC technique labels proteins with either natural “light” or nonradioactive, stable isotope-containing “heavy” amino-acids using the natural metabolic machinery of the cell (typically ^13^C_6_^15^N_2_-Lys (8.0142 Da) and ^13^C_6_^14^N_4_-Arg (10.00827 Da), resp.). When “light” and “heavy” amino-acid-labeled cells are mixed, those cell populations remain distinguishable by mass-spectrometry. Therefore, protein abundances can be determined from the relative mass-spectrometry signal intensities [91, 92]. 

 Uses of the SILAC technique include studying differential protein expression and identifying biomarkers and drug targets in pancreatic [93] and breast cancer [94, 95]. Bose and colleagues used the SILAC method to analyze ErbB2/Her2 signal transduction pathways and the effect of a tyrosine kinase inhibitor, PD168393 [94]. This study led to the identification of important phosphoproteins including many known Her2 and EGFR signaling proteins, as well as previously unidentified Her2 signaling proteins, such as Stat1, Dok1, and *δ*-catenin, providing valuable leads for designing optimal future therapies in Her2-related breast cancer. Another recent study used this method in breast cancer cells treated with suberoylanilide hydroxamic acid (Vorinostat), a histone deacetylase (HDAC) inhibitor, and revealed changes in the expression of transcription factors, regulators, chaperones, cell structure proteins and glycolytic enzymes, further defining the function of the HDAC inhibitor in breast cancer [96]. These studies demonstrated that the SILAC is a powerful method for investigating the dynamics of protein abundance in signaling networks.

 However, the limitation of this technology is that it requires full metabolic labeling of the whole proteome; it is, therefore, thought to be suitable only for analyzing cell culture not human tissue or body fluid samples [92]. Mann and his colleagues have recently developed a “super-SILAC” mixture, which combines five SILAC-labeled cell lines with human carcinoma tissue, generating sufficient amounts of isotopically labeled peptides to serve as internal standards for mass-spectrometry-based analysis [92]. It seems that super-SILAC has opened a new avenue in quantitative proteomics and it may hold considerable promise for the future study of the cancer proteome.

## 14. Matrix-Assisted Laser Desorption Ionization-Imaging Mass Spectrometry (MALDI-IMS)

Developments of matrix desorption and ionization techniques such as matrix-assisted laser desorption ionization-mass-spectrometry (MALDI-MS) [97, 98] and electrospray ionization-mass-spectrometry (ESI-MS) [99] have clearly revolutionized protein science. These improvements offer higher accuracy and sensitivity of the protein mass measurement. MALDI-imaging mass-spectrometry (MALDI-IMS) is a new imaging method based on mass-spectrometry, allowing the direct visualization of peptides, proteins, lipids, and metabolites as well as other low mass small-molecules on fresh frozen or fixed paraffin-embedded tissue sections [100]. 

The invention of MALDI-IMS was a major breakthrough by making it possible to study the localization and abundance of molecules without multiple steps of sample preparation. In addition, fresh frozen tissue samples are used in this assay. Briefly, matrix is uniformly applied to the tissue section, which has already been mounted and sliced, and then the scanning is carried out in raster fashion over thin tissue sections. Each pixel contains information of a full mass spectrum, thus, the molecular profile of the proteomic content of the tissue is available [101]. One very striking finding resulting from this technology is that the aberrant changes in protein expression within kidney tumors are also detected in the normal tissue that surrounds the tumor [102, 103].

Since MALDI-IMS can produce molecular protein signatures of both healthy and diseased tissues, it has successfully led to the identification of diagnostic, prognostic, and therapeutic protein biomarkers and produced a novel classification of diseases [104, 105]. Proteomic profiling using MALDI-IMS has been applied to multiple types of diseased tissues, including human nonsmall-cell lung tumors [106], gliomas [104], prostate [107], breast [108], and ovarian cancer [109]. In addition, molecular signatures provided by MALDI-IMS applied to study of therapeutic response to HER2 receptor inhibitors OSI-774 and trastuzumab/Herceptin [110], tumor grading, and prediction of patient survival [100]. Recent data reported the ability of a proteomic signature to accurately define HER2-positive from HER2-negative tissues [108]. Collectively, MALDI-IMS using frozen or fixed paraffin-embedded tissues has shown unique advantages and could provide a better understanding of cancer onset and progression and a new strategy for biomarker discovery.

## 15. Activity-Based Protein Profiling (ABPP)

Activity-based protein profiling (ABPP), a powerful resource for functional proteomics, was first invented by Dr. James C. Powers in the early 1990s and adopted and developed by Dr. Benjamin F. Cravatt III and other researchers to identify specific activities in proteome samples [111]. ABPP employs active site-directed, small-molecule-based covalent probes to directly determine the functional state of large numbers of enzymes in native biological systems [112]. The probes for this assay, called activity-based probes, must have both a reactive group to bind in enzyme activation sites and a reporter tag, such as a fluorophore, that is required for their detection [113]. As examples of this, activity-based probes have been successfully developed for many enzyme classes, including histone deacetylases [114, 115], caspases [116], cytochrome P450s [117], metalloproteinases [118], proteases [119, 120], kinases [121–123], phosphatases [124], glycosidases [125], and oxidoreductases [126–128]. 

 For *in situ* or *in vivo* labeling, the activity-based probes are altered through substituting the reporter tag with a handle, such as an alkyne or azide for use with the Huisgen 1,3-dipolar cycloaddition (click chemistry) [129, 130]. *In vitro* activity-based fluorescent probes can provide the dynamics of enzyme activation over time, with the caveat of photo bleaching in live cell conditions. Since the handle groups will not react intracellularly, this is a clean method to probe for inhibitors of enzyme active sites. The novelty in this approach *in vivo *is the ability to visualize whether an inhibitor is working in a small animal model. Mechanistic studies have confirmed that these probes can distinguish active enzyme from their zymogen or inhibitor-bound forms [128]. 

 There have been many attempts to discover cancer-relevant enzymes and new enzyme inhibitors by using ABPP platforms. For example, using ABPs for the analysis of cysteine proteases, Joyce and colleagues found that cysteine cathepsin proteases are upregulated in HPV-induced cervical carcinomas [119]. Rolén et al. used this strategy to profile the activities of individual ubiquitin-specific proteases (USPs) in biopsies of HPV carrying cervical carcinoma and adjacent normal tissue, and as a result, they discovered that ubiquitin-carboxy hydrolase-L3 (UCHL3) and UCH37 are increased in HPV-positive tumors [131], which encourages further consideration of its use as therapeutic targets in cervical cancer. Comparative ABPP analysis of an *in-vivo*-derived variant of the human breast cancer cell line MDA-MB-231, termed 231MFP, and parental MDA-MB-231 cells revealed dramatic alterations in enzyme activities such as upregulation of the serine proteases tissue-type (tPA) and urokinase-type (uPA) plasminogen activators in the 231MFP cell line [132], highlighting the importance of an ABPP for identifying and characterizing the protein signatures. Wright and colleagues demonstrated the potential clinical use of ABPs in drug discovery for cancer-related enzymes. They used cytochrome-p450s- (CYP450s-) directed ABPs to profile the effects of CYP450 activity on aromatase inhibitors, which are used in breast cancer treatment. This led to the identification of the aromatase inhibitor, anastrozole, as an inducer of CYP1A2 activity in breast cancer [117]. Based on these findings, the technique appears to be exceptional for cancer research given the aberrant functions many of these enzyme classes have in cancer.

 In recent years, ABPP has been combined with mass-spectrometry-based platforms such as tandem MS and liquid chromatography MS (LC/MS) to achieve great coverage of the proteome [133–135]. For example, the integration of ABPP with a liquid chromatography- (LC-) MS-based platform, termed Multidimensional Protein Identification Technology (MudPIT), identified many enzyme activities in breast cancer samples, and especially three enzymes were significantly upregulated in ER-/PR- breast cancers compared to either ER+/PR+ or normal tissues [136]: (i) KIAA1363, a serine hydrolase that is elevated in aggressive breast and ovarian cancers [137]; (ii) fibroblast activation protein (FAP/Seprase), a cell-surface serine protease, promoting growths of breast cancer in part, through enhanced angiogenesis [138]; and (iii) platelet-activating factor acetylhydrolase 2 (PAF-AH2), a lipase that degrades the endogenous signaling molecule PAF [136, 139]. Therefore, the combination of ABPP and mass-spectrometry-based proteomic profiling may provide systematic and comprehensive information for target identification, target validation, and drug discovery and allow the integration of individual enzyme activities into the larger metabolic networks of cancer cells.

## 16. Reverse Phase Protein Array (RPPA)

Posttranslational modification plays a pivotal role in cellular signaling pathways, and one prominent biochemical role is played by phosphorylation, indicating the need for technologies capable of detecting dynamic cellular changes in the proteome. Although Western blotting has been a laboratory staple for decades and is capable of detecting protein phosphorylation, the limitations of this technique preclude its incorporation into a clinical diagnostic setting for examining patient specimens. Thus, the high-throughput, novel, proteomic approach reverse phase protein array (RPPA) was developed to quantify signaling pathway activation using protein phosphorylation-specific antibodies among a large number of biological specimens, and RPPA was validated [140–142]. The format allows measurement of protein expression levels using antibodies on multiple biological samples simultaneously in a quantitative manner. 

Many studies have shown the ability of RPPA to simultaneously monitor multiple signaling pathways, allowing rapid screening of potential molecular tumor markers and targets [143, 144]. Dr. Gordon B. Mills and his group have applied RPPA technique to profile and validate key signaling networks dysregulated in women's cancers, including ovarian and breast cancer, and these studies provide novel insights into the diverse cell signaling-based mechanisms underlying cancer progression [145–149]. Bartholomeusz and colleagues used RPPAs to measure PEA-15 (also called PED), which is believed to be a novel regulator of the ERK/MAP kinase pathway, in 320 human breast cancers and discovered that low expression of PEA-15 is correlated with high nuclear grade and negative hormone receptor status [150], suggesting its therapeutic potential as a druggable target in breast cancer.

The antibody-based proteomics technique is a powerful approach to functionally explore the human proteome using specific antibodies and thus enable the generation of a comprehensive proteomic network [151]. The major advantage of RPPA is that it requires only miniscule sample volume for protein detection, some suggesting that ~20 cells provide a sufficient amount [152–154]. Therefore, very limited amounts of sample, such as from a biopsy, could also be analyzed for biomarker discovery and/or other clinical diagnostics using this method.

Another major advantage of RPPA is that it can be used for monitoring the protein dynamics over time. The whole-cell lysates are immobilized in individual spots on an array platform in a serial dilution curve, and then each array can be probed with a specific antibody that can be detected by chemiluminescence, fluorescence, or colorimetric assays. The array slide is scanned, and using the image, the visual spots are quantified using specialized software designed for this purpose. Multiplexing is carried out by probing multiple protein arrays spotted with the same lysate and using different antibodies [155]. So far, RPPA has shown great potential for clinical application; however, the variability and comparability of staining between arrays represents a major challenge to solve. Another disadvantage of using RPPA is that it requires very-high-quality antibodies that have been validated through traditional immunoblotting, which is a time-consuming bottleneck for this assay.

## 17. ELISA and Multiplex Assay

Although the enzyme-linked immunosorbent assay (ELISA) is a “gold standard” for protein detection in biological samples, it is quickly being updated and replaced by improved techniques. The ELISA technique has been used in laboratories for some time, even appearing in greatly dated biological textbooks, making it well understood and utilized by many as a complement or substitute for Western blotting. Although ELISA is reliable, quantitative, and accurate, classical ELISA uses a single antigen detection method and often requires a relatively large volume of a biological sample, which may make repeat testing difficult. Furthermore, it is not often the case that large sample amounts are available, particularly when replicates are necessary for accuracy. Even if replicates are possible, a measurement from a heterogeneous population of cells may be obscured using this method if a subpopulation dominates the protein pool. Nevertheless, its historical nature ensures that many of the biological tests performed in diagnostic labs today are using ELISA. For example, there are many different types of ELISA test kits commercially available for cervical cancer screening such as Cervatec ELISA kit to detect p16^INK4A^ and human HPV ELISA kits to detect IgG or IgM. 

Multiplex assays have been developed to overcome challenges encountered in the classical ELISA, such as large sample volume format, extensive time requirement, labor-intensity, and the issue of quantifying multiple antigens in a single sample [151]. The quality of the antibody and its specificity for the target antigen remain a crucial determinant in these assays and can frustrate even the most well-intentioned scientists with otherwise infallible logic and perfectly designed epitopes. Currently, flow cytometry-, chemiluminescence-, and electrochemiluminescence-based multiplex assays are commercially available [156]. Flow cytometry multiplex arrays (also known as bead-based multiplex assays) are currently a commonly used format. Each bead set is coated with a specific capture antibody for a single antigen, and fluorescence or streptavidin-labeled antibodies bind to the specific capture antibody complex on the bead set, which can be detected by flow cytometry. The cytometric bead array (CBA) system (BD Biosciences), the Luminex multianalyte profiling (xMAP) technology (Luminex), and MultiBead multiplex assay (Enzo Life Science) provides a flow cytometry-based multiplex assay. 

Multiplex arrays have several advantages that improve upon the challenges with ELISA, in particular that they require far less sample volume per antigen, can be completed within a shorter time, are more cost efficient per antigen, and have the ability to reliably detect different proteins across a broad dynamic range of concentrations [151, 156]. Multiplex ELISA (Quansys Biosciences) and Proteome Profiler Arrays (R&D Systems) provide a 96-well microplate coated with multiple, specific capture antibodies, followed by chemiluminescent detection. MSD Multi-Array (Meso Scale Discovery) employs electrochemiluminescence technology for detection. They array the capture antibody on a microplate with electrodes integrated into the bottom of the plate, followed by detection with an electrochemiluminescent tag. Studies examining the effect of the PI3K inhibitor LY294002 on human xenografts demonstrated the multiplex assays' effectiveness and reliability to measure pharmacodynamic responses [157]. Through these breakthrough technologies and applications, multiplex assays for the detection of biomarkers are becoming popular in preclinical and clinical diagnostic research.

## 18. Tissue Microarray and Automated Quantitative Assessment of Immunofluorescence (TMA-AQUA)

Cancer biomarker studies using the combination of tissue microarray and automated quantitative assessment of immunofluorescence (TMA-AQUA) have been successfully applied for various types of human cancer [158]. TMA is a high-throughput pathology platform for molecular profiling of tumor specimens, principally using immunohistochemistry method [159], and has become a powerful tool for cancer biomarker discovery over the past decade [160, 161]. Identifying and scoring cancer markers help researchers to characterize the tumor and predict disease progression, however, the current method for immunohistochemistry scoring is labor-intensive, has inherent limitations to quantification and has the potential to introduce bias into the results [162]. Therefore, the need for improvement of the quality, reproducibility, and accuracy of immunohistochemistry is obvious. Various automated image analysis software are currently available, and several groups have demonstrated that automated IHC scoring systems offer time efficiency, better accuracy, good reproducibility, and higher quality data compared to manual analysis [163–166].

The HistoRx AQUA platform, a well-established, automated, fluorescence-based immunohistochemistry image analysis, identifies tumor cells using tumor-specific proteins such as cytokeratin. Doing this creates an interest region and allows the quantitative assessment of protein expression in formalin-fixed paraffin-embedded tissue samples [167]. The output of the analysis (called the “AQUA score”) can be correlated with other parameters such as disease detection, progression, or response to therapy. The AQUA system allows for high-throughput and high-resolution analysis of TMA, therefore, the TMA-AQUA can serve as an effective discovery tool and will help to advance personalized medicine by identifying and validating new biomarker and drug targets.

Several studies demonstrated that AQUA is capable of measuring protein expression on histological specimens obtained from various types of tumor with good accuracy and reproducibility, possibly leading to better clinical outcomes [168–171]. Aitken and colleagues used TMA-AQUA platform for quantitative analysis of changes in ER, PR, and Her2 expression in invasive primary breast carcinomas and paired lymph nodes and demonstrated that this new method could be a more accurate measurement for conferring optimal benefits to adjuvant therapy [172]. Harigopal and colleagues used this method to investigate the prognostic significance of AIB1, TIF2, and NCoR protein expression in breast cancer. As a result, high expression of AIB1 was strongly correlated with poor patient survival, suggesting its potential use as a prognostic and predictive marker in breast cancer [173].

## 19. Summary

Large-scale genomic studies, such as the Cancer Genome Atlas (TCGA) project funded by the National Human Genome Research Institute (NHGRI) and the National Cancer Institute (NCI), are identifying numerous cancer susceptibility genes and profiling gene expression and have made great strides in highlighting the molecular basis of cancer. Although transcriptome and genome studies are certainly worthwhile, these cannot alone capture the view of cancer dynamics, without the incorporation of proteomics. The advancement of proteomic technologies has ushered in a new paradigm for highly integrative cancer research in many ways, and the integration with other “OMICS” technologies now provides complementary information to better understand the genetic nature of cancer. New and emerging proteomics technologies are very promising and now readily feasible for clinical applications including the identification of novel biomarkers, monitoring therapy response, and disease progression, delivering on the great promise of personalized cancer medicine. Although proteomics still have challenges that need to be overcome, these approaches will catalyze the development of platforms for personalized medicine.

## Figures and Tables

**Table 1:** Age-adjusted SEER incidence rates by age at diagnosis/death.

	Statistical probability of diagnosis				
Woman's age	<20	20–49	50–64	65–74	>75
Breast cancer	—	72.5	270	416	414
Cervical cancer	—	8.3	9.6	12	9.2
Colon cancer	—	10	60	152	270
Corpus and uterine cancer	—	9.4	66	95	73
Lung cancer	—	6.7	76	265	328
Melanoma	0.79	16	29	41	46
Ovarian cancer	0.42	5.8	27.9	41	49
Pancreatic cancer	—	1.4	14	45	79
Thyroid cancer	1.2	24	30	28	16
Vaginal cancer	—	—	1.2	2.5	3.3
Vulvar cancer	—	0.92	3.5	7.1	13

Informational source: Altekruse SF, Kosary CL, Krapcho M, et al. SEER Cancer Statistics Review, 1975–2007. Bethesda, MD: National Cancer Institute, 2010.

“Incidence source: SEER 9 areas (San Francisco, Connecticut, Detroit, Hawaii, Iowa, New Mexico, Seattle, Utah, and Atlanta). Rates are per 100,000 and are age-adjusted to the 2000 USA Std Population.”

— Statistics not displayed, <16 cases.

**Table 2:** Tests for risk assessment, treatment, or outcome in breast cancer.

Breast cancer test	Information obtained
Adjuvant! Online	10-year overall survival
Gail risk model	Estimates the risk for developing breast cancer
MammaPrint	Relapse and metastasis
Mammostrat	Clinical outcome and overall survival
Onco*type* DX	Recurrence and adjuvant therapy recommendation
St. Gallen Consensus	Chemotherapy recommendation

**Table 3:** HPV types and associated diseases.

Disease	HPV types
Genital warts	6, 11
Flat condyloma	6, 11,16,18,31
Cervical cancer	16, 18 (strong association)
	31, 33, 35, 45, 51, 52, 56
	(moderate association)
	6, 11, 42, 43, 44 (weak association)
Vulvar intraepithelial neoplasia	16
Oral focal epithelial hyperplasia	13, 32
Oral papillomas	6, 7, 11, 16, 32
Oropharyngeal cancer	16
Laryngeal papillomatosis	6, 11

## References

[1] Jemal A (2010). Cancer statistics, 2010. *CA: A Cancer Journal for Clinicians*.

[2] Harlan LC (2010). Breast cancer in men in the United States: a population-based study of diagnosis, treatment, and survival. *Cancer*.

[3] National Cancer Institute, D., Surveillance 
Research Program, Cancer Statistics Branch, Surveillance, Epidemiology, and End Results (SEER) Program http://www.seer.cancer.gov/.

[4] Kuerer HM, Albarracin CT, Yang WT (2009). Ductal carcinoma in situ: state of the science and roadmap to advance the field. *Journal of Clinical Oncology*.

[5] Whitman G, Kushwaha A (2008). Mammography, magnetic resonance imaging of the breast, and radionuclide imaging of the breast. *Breast Cancer*.

[6] Shin SJ, Rosen PP (2002). Excisional biopsy should be performed if lobular carcinoma in situ is seen on needle core biopsy. *Archives of Pathology and Laboratory Medicine*.

[7] Carder PJ (2010). Screen-detected pleomorphic lobular carcinoma in situ (PLCIS): risk of concurrent invasive malignancy following a core biopsy diagnosis. *Histopathology*.

[8] (2009). Screening for breast cancer: U.S. Preventive Services Task force recommendation statement. *Annals of Internal Medicine*.

[9] Galea MH, Blamey RW, Elston CE, Ellis IO (1992). The Nottingham prognostic index in primary breast cancer. *Breast Cancer Research and Treatment*.

[10] Oakman C, Santarpia L, Di Leo A (2010). Breast cancer assessment tools and optimizing adjuvant therapy. *Nature Reviews Clinical Oncology*.

[11] Bartlett JM (2010). Mammostrat as a tool to stratify breast cancer patients at risk of recurrence during endocrine therapy. *Breast Cancer Research*.

[12] NCT00310180.

[13] Tavassoli FA (2010). Correlation between gene expression profiling-based molecular and morphologic classification of breast cancer. *International Journal of Surgical Pathology*.

[14] Perou CM, Sørile T, Eisen MB (2000). Molecular portraits of human breast tumours. *Nature*.

[15] Sørlie T, Perou CM, Tibshirani R (2001). Gene expression patterns of breast carcinomas distinguish tumor subclasses with clinical implications. *Proceedings of the National Academy of Sciences of the United States of America*.

[16] Bergamaschi A, Tagliabue E, Sørlie T (2008). Extracellular matrix signature identifies breast cancer subgroups with different clinical outcome. *Journal of Pathology*.

[17] Priestman T (2007). *Cancer Chemotherapy in Clinical Practice*.

[18] Jordan VC, Morrow M (1999). Tamoxifen, raloxifene, and the prevention of breast cancer. *Endocrine Reviews*.

[19] Pinder M, Buzdar A (2008). Endocrine therapy for breast cancer. *Breast Cancer*.

[20] Johnston SR, Dowsett M (2003). Aromatase inhibitors for breast cancer: lessons from the laboratory. *Nature Reviews Cancer*.

[21] Banerjee S, Smith IE (2010). Management of small HER2-positive breast cancers. *The Lancet Oncology*.

[22] Roche H-L NCT00545688.

[23] Maxmen A (2010). Beyond PARP inhibitors: agents in pipelines target DNA repair mechanisms. *National Cancer Institute*.

[24] O’Shaughnessy J (2011). Iniparib plus chemotherapy in metastatic triple-negative breast cancer. *The New England Journal of Medicine*.

[25] Green M, Hortobagyi G (2008). Chemotherapy for breast cancer. *Breast Cancer*.

[26] Chu E, DeVita VT (2008). *Physicians’ Cancer Chemotherapy Drug Manual*.

[27] Sankaranarayanan R, Ferlay J (2006). Worldwide burden of gynaecological cancer: the size of the problem. *Best Practice & Research Clinical Obstetrics & Gynaecology*.

[28] Walboomers JM, Jacobs MV, Manos MM (1999). Human papillomavirus is a necessary cause of invasive cervical cancer worldwide. *Journal of Pathology*.

[29] zur Hausen H (2002). Papillomaviruses and cancer: from basic studies to clinical application. *Nature Reviews Cancer*.

[30] Yim EK, Park JS (2007). Biomarkers in cervical cancer. *Biomarker Insights*.

[31] Hu D, Goldie S (2008). The economic burden of noncervical human papillomavirus disease in the United States. *American Journal of Obstetrics and Gynecology*.

[32] Cattani P, Siddu A, D’Onghia S (2009). RNA (E6 and E7) assays versus DNA (E6 and E7) assays for risk evaluation for women infected with human papillomavirus. *Journal of Clinical Microbiology*.

[33] Schweizer J (2010). Feasibility study of a human papillomavirus E6 oncoprotein test for diagnosis of cervical precancer and cancer. *Journal of Clinical Microbiology*.

[34] Klaes R, Friedrich T, Spitkovsky D (2001). Overexpression of p16(INK4A) as a specific marker for dysplastic and neoplastic epithelial cells of the cervix uteri. *International Journal of Cancer*.

[35] Dray M, Russell P, Dalrymple C (2005). p16(INK4a) as a complementary marker of high-grade intraepithelial lesions of the uterine cervix. I: experience with squamous lesions in 189 consecutive cervical biopsies. *Pathology*.

[36] Lakshmi S, Rema P, Somanathan T (2009). P16(INK4a) is a surrogate marker for high-risk and malignant cervical lesions in the presence of human papillomavirus. *Pathobiology*.

[37] Agoff SN, Lin P, Morihara J, Mao C, Kiviat NB, Koutsky LA (2003). p16(INK4a) expression correlates with degree of cervical neoplasia: a comparison with Ki-67 expression and detection of high-risk HPV types. *Modern Pathology*.

[38] Negri G, Vittadello F, Romano F (2004). P16(INK4a) expression and progression risk of low-grade intraepithelial neoplasia of the cervix uteri. *Virchows Archiv*.

[39] Samarawardana P (2010). p16(INK4a) is superior to high-risk human papillomavirus testing in cervical cytology for the prediction of underlying high-grade dysplasia. *Cancer Cytopathology*.

[40] Kato H, Torigoe T (1977). Radioimmunoassay for tumor antigen of human cervical squamous cell carcinoma. *Cancer*.

[41] Duk JM, Groenier KH, De Bruijn HWA (1996). Pretreatment serum squamous cell carcinoma antigen: a newly identified prognostic factor in early-stage cervical carcinoma. *Journal of Clinical Oncology*.

[42] Bolli JA (1994). Squamous cell carcinoma antigen: clinical utility in squamous cell carcinoma of the uterine cervix. *Gynecologic Oncology*.

[43] Brioschi PA, Bischof P, Delafosse C, Krauer F (1991). Squamous-cell carcinoma antigen (SCC-A) values related to clinical outcome of pre-invasive and invasive cervical carcinoma. *International Journal of Cancer*.

[44] Hong JH, Tsai CS, Chang JT (1998). The prognostic significance of pre- and posttreatment SCC levels in patients with squamous cell carcinoma of the cervix treated by radiotherapy. *International Journal of Radiation Oncology Biology Physics*.

[45] Ross W, Hall PA (1995). Ki67: from antibody to molecule to understanding?. *Clinical Molecular Pathology*.

[46] al-Saleh W, Delvenne P, Greimers R, Fridman V, Doyen J, Boniver J (1995). Assessment of Ki-67 antigen immunostaining in squamous intraepithelial lesions of the uterine cervix: correlation with the histologic grade and human papillomavirus type. *American Journal of Clinical Pathology*.

[47] Kruse AJ, Baak JPA, De Bruin PC (2001). Ki-67 immunoquantitation in cervical intraepithelial neoplasia (CIN): a sensitive marker for grading. *Journal of Pathology*.

[48] Kruse AJ, Baak JPA, Janssen EA (2004). Ki67 predicts progression in early CIN: validation of a multivariate progression-risk model. *Cellular Oncology*.

[49] Kearsey SE, Labib K (1998). MCM proteins: evolution, properties, and role in DNA replication. *Biochimica et Biophysica Acta*.

[50] Gonzalez MA, Tachibana KEK, Laskey RA, Coleman N (2005). Control of DNA replication and its potential clinical exploitation. *Nature Reviews Cancer*.

[51] Kearsey SE, Maiorano D, Holmes EC, Todorov IT (1996). The role of MCM proteins in the cell cycle control of genome duplication. *Bioessays*.

[52] Maiorano D, Van Assendelft GB, Kearsey SE (1996). Fission yeast cdc21, a member of the MCM protein family, is required for onset of S phase and is located in the nucleus throughout the cell cycle. *The EMBO Journal*.

[53] Williams GH, Romanowski P, Morris L (1998). Improved cervical smear assessment using antibodies against proteins that regulate DNA replication. *Proceedings of the National Academy of Sciences of the United States of America*.

[54] Murphy N, Ring M, Heffron CCBB (2005). p16INK4A, CDC6, and MCM5: predictive biomarkers in cervical preinvasive neoplasia and cervical cancer. *Journal of Clinical Pathology*.

[55] Freeman A, Morris LS, Mills AD (1999). Minichromosome maintenance proteins as biological markers of dysplasia and malignancy. *Clinical Cancer Research*.

[56] Brake T, Connor JP, Petereit DG, Lambert PF (2003). Comparative analysis of cervical cancer in women and in a human papillomavirus-transgenic mouse model: identification of minichromosome maintenance protein 7 as an informative biomarker for human cervical cancer. *Cancer Research*.

[57] Hannemann J, Kristel P, Van Tinteren H (2006). Molecular subtypes of breast cancer and amplification of topoisomerase IIalpha: predictive role in dose intensive adjuvant chemotherapy. *British Journal of Cancer*.

[58] Chen Y, Miller C, Mosher R (2003). Identification of cervical cancer markers by cDNA and tissue microarrays. *Cancer Research*.

[59] Branca M (2006). Over-expression of topoisomerase IIalpha is related to the grade of cervical intraepithelial neoplasia (CIN) and high-risk human papillomavirus (HPV), but does not predict prognosis in cervical cancer or HPV clearance after cone treatment. *International Journal of Gynecological Pathology*.

[60] Pinto AP (2008). Biomarker (ProEx C, p16(INK4A), and MiB-1) distinction of high-grade squamous intraepithelial lesion from its mimics. *Modern Pathology*.

[61] Fernandez MA, Allen JD, Mistry R, Kahn JA (2010). Integrating clinical, community, and policy perspectives on human papillomavirus vaccination. *Annual Review of Public Health*.

[62] DiSaia PJ, Creasman WT (2007). *Clinical Gynecologic Oncology*.

[63] Diamandis EP (2010). Cancer biomarkers: can we turn recent failures into success?. *Journal of the National Cancer Institute*.

[64] Donach M, Yu Y, Artioli G (2010). Combined use of biomarkers for detection of ovarian cancer in high-risk women. *Tumour Biology*.

[65] Yurkovetsky Z, Skates S, Lomakin A (2010). Development of a multimarker assay for early detection of ovarian cancer. *Journal of Clinical Oncology*.

[66] Bast RC, Feeney M, Lazarus H (1981). Reactivity of a monoclonal antibody with human ovarian carcinoma. *Journal of Clinical Investigation*.

[67] Guarneri V (2011). Timing for starting second-line therapy in recurrent ovarian cancer. *Expert Review of Anticancer Therapy*.

[68] Chitale R (2009). Monitoring ovarian cancer: CA125 trial stirs controversy. *Journal of the National Cancer Institute*.

[69] Andriole GL, Berg CD, Crawford ED (2009). Mortality results from a randomized prostate-cancer screening trial. *New England Journal of Medicine*.

[70] NCT00539162.

[71] Sussman L (2010). Ovarian cancer study a life-saver. *Conquest*.

[72] Jacobs I (2011). Sensitivity of transvaginal ultrasound screening for endometrial cancer in postmenopausal women: a case-control study within the UKCTOCS cohort. *The Lancet Oncology*.

[73] http://www.ova-1.com/.

[74] Helistrom I, Raycraft J, Hayden-Ledbetter M (2003). The HE4 (WFDC2) protein is a biomarker for ovarian carcinoma. *Cancer Research*.

[75] Hellstrom I, Hellstrom KE (2008). SMRP and HE4 as biomarkers for ovarian carcinoma when used alone and in combination with CA125 and/or each other. *Advances in Experimental Medicine and Biology*.

[76] Moore RG, McMeekin DS, Brown AK (2009). A novel multiple marker bioassay utilizing HE4 and CA125 for the prediction of ovarian cancer in patients with a pelvic mass. *Gynecologic Oncology*.

[77] Bruckner HW, Cohen CJ, Wallach RC (1978). Treatment of advanced ovarian cancer with cis-dichlorodiammineplatinum(II): poor-risk patients with intensive prior therapy. *Cancer Treatment Reports*.

[78] Cavalli F (1976). [First therapeutic experience with cis-platinum(II)diamindichloride (NSC 119875) in metastasizing ovarian and testicular carcinoma]. *Schweizerische Medizinische Wochenschrift*.

[79] Thigpen JT (1983). Cis-platinum in the treatment of advanced or recurrent adenocarcinoma of the ovary. A phase II study of the Gynecologic Oncology Group. *American Journal of Clinical Oncology*.

[80] Markman M, Rowinsky E, Hakes T (1992). Phase I trial of intraperitoneal taxol: a Gynecologic Oncology Group study. *Journal of Clinical Oncology*.

[81] Einzig AI, Wiernik PH, Sasloff J, Runowicz CD, Goldberg GL (1992). Phase II study and long-term follow-up of patients treated with taxol for advanced ovarian adenocarcinoma. *Journal of Clinical Oncology*.

[82] Gunby P (1992). National cancer institute making more taxol available for refractory ovarian, breast cancers. *Journal of the American Medical Association*.

[83] DeFuria MD, Horovitz Z (1993). Taxol commercial supply strategy. *Journal of the National Cancer Institute*.

[84] Murph M, Tanaka T, Pang J (2007). Liquid chromatography mass spectrometry for quantifying plasma lysophospholipids: potential biomarkers for cancer diagnosis. *Methods in Enzymology*.

[85] Tanaka T, Tsutsui H, Hirano K, Koike T, Tokumura A, Satouchi K (2004). Quantitative analysis of lysophosphatidic acid by time-of-flight mass spectrometry using a phosphate-capture molecule. *Journal of Lipid Research*.

[86] Altman MK, Gopal V, Jia W (2010). Targeting melanoma growth and viability reveals dualistic functionality of the phosphonothionate analogue of carba cyclic phosphatidic acid. *Molecular Cancer*.

[87] Roepstorff P (1997). Mass spectrometry in protein studies from genome to function. *Current Opinion in Biotechnology*.

[88] Lahm HW, Langen H (2000). Mass spectrometry: a tool for the identification of proteins separated by gels. *Electrophoresis*.

[89] Godovac-Zimmermann J, Brown LR (2001). Perspectives for mass spectrumetry and functional proteomics. *Mass Spectrometry Reviews*.

[90] Mann M (2006). Functional and quantitative proteomics using SILAC. *Nature Reviews Molecular Cell Biology*.

[91] Ong SE, Mann M (2007). A practical recipe for stable isotope labeling by amino acids in cell culture (SILAC). *Nature Protocols*.

[92] Geiger T, Cox J, Ostasiewicz P, Wisniewski JR, Mann M (2010). Super-SILAC mix for quantitative proteomics of human tumor tissue. *Nature Methods*.

[93] Gronborg M, Kristiansen TZ, Iwahori A (2006). Biomarker discovery from pancreatic cancer secretome using a differential proteomic approach. *Molecular and Cellular Proteomics*.

[94] Bose R, Molina H, Scott Patterson A (2006). Phosphoproteomic analysis of Her2/neu signaling and inhibition. *Proceedings of the National Academy of Sciences of the United States of America*.

[95] Liang X, Zhao J, Hajivandi M (2006). Quantification of membrane and membrane-bound proteins in normal and malignant breast cancer cells isolated from the same patient with primary breast carcinoma. *Journal of Proteome Research*.

[96] Zhou Q, Chaerkady R, Shaw PG, Kensler TW, Pandey A, Davidson NE (2010). Screening for therapeutic targets of vorinostat by SILAC-based proteomic analysis in human breast cancer cells. *Proteomics*.

[97] Karas M, Hillenkamp F (1988). Laser desorption ionization of proteins with molecular masses exceeding 10 000 daltons. *Analytical Chemistry*.

[98] Covey TR, Huang EC, Henion JD (1991). Structural characterization of protein tryptic peptides via liquid chromatography/mass spectrometry and collision-induced dissociation of their doubly charged molecular ions. *Analytical Chemistry*.

[99] Fenn JB, Mann M, Meng CK, Wong SF, Whitehouse CM (1989). Electrospray ionization for mass spectrometry of large biomolecules. *Science*.

[100] Schwamborn K, Caprioli RM (2010). Molecular imaging by mass spectrometry—looking beyond classical histology. *Nature Reviews Cancer*.

[101] Chaurand P, Schwartz SA, Reyzer ML, Caprioli RM (2005). Imaging mass spectrometry: principles and potentials. *Toxicologic Pathology*.

[102] Herring KD, Oppenheimer SR, Caprioli RM (2007). Direct tissue analysis by matrix-assisted laser desorption ionization mass spectrometry: application to kidney biology. *Seminars in Nephrology*.

[103] Oppenheimer SR, Mi D, Sanders ME, Caprioli RM (2010). Molecular analysis of tumor margins by MALDI mass spectrometry in renal carcinoma. *Journal of Proteome Research*.

[104] Stoeckli M, Chaurand P, Hallahan DE, Caprioli RM (2001). Imaging mass spectrometry: a new technology for the analysis of protein expression in mammalian tissues. *Nature Medicine*.

[105] Seeley EH, Caprioli RM (2008). Molecular imaging of proteins in tissues by mass spectrometry. *Proceedings of the National Academy of Sciences of the United States of America*.

[106] Yanagisawa K, Shyr Y, Xu BJ (2003). Proteomic patterns of tumour subsets in non-small-cell lung cancer. *Lancet*.

[107] Schwamborn K, Krieg RC, Reska M, Jakse G, Knuechel R, Wellmann A (2007). Identifying prostate carcinoma by MALDI-Imaging. *International Journal of Molecular Medicine*.

[108] Rauser S, Marquardt C, Balluff B (2010). Classification of HER2 receptor status in breast cancer tissues by MALDI imaging mass spectrometry. *Journal of Proteome Research*.

[109] Lemaire R, Menguellet SA, Stauber J (2007). Specific MALDI imaging and profiling for biomarker hunting and validation: fragment of the 11S proteasome activator complex, reg alpha fragment, is a new potential ovary cancer biomarker. *Journal of Proteome Research*.

[110] Reyzer ML, Caldwell RL, Dugger TC (2004). Early changes in protein expression detected by mass spectrometry predict tumor response to molecular therapeutics. *Cancer Research*.

[111] Liu Y, Patricelli MP, Cravatt BF (1999). Activity-based protein profiling: the serine hydrolases. *Proceedings of the National Academy of Sciences of the United States of America*.

[112] Simon GM, Cravatt BF (2010). Activity-based proteomics of enzyme superfamilies: serine hydrolases as a case study. *Journal of Biological Chemistry*.

[113] Nomura DK, Dix MM, Cravatt BF (2010). Activity-based protein profiling for biochemical pathway discovery in cancer. *Nature Reviews Cancer*.

[114] Salisbury CM, Cravatt BF (2007). Activity-based probes for proteomic profiling of histone deacetylase complexes. *Proceedings of the National Academy of Sciences of the United States of America*.

[115] Salisbury CM, Cravatt BF (2008). Optimization of activity-based probes for proteomic profiling of histone deacetylase complexes. *Journal of the American Chemical Society*.

[116] Edgington LE, Berger AB, Blum G (2009). Noninvasive optical imaging of apoptosis by caspase-targeted activity-based probes. *Nature Medicine*.

[117] Wright AT, Song JD, Cravatt BF (2009). A suite of activity-based probes for human cytochrome P450 enzymes. *Journal of the American Chemical Society*.

[118] Saghatelian A, Jessani N, Joseph A, Humphrey M, Cravatt BF (2004). Activity-based probes for the proteomic profiling of metalloproteases. *Proceedings of the National Academy of Sciences of the United States of America*.

[119] Joyce JA, Baruch A, Chehade K (2004). Cathepsin cysteine proteases are effectors of invasive growth and angiogenesis during multistage tumorigenesis. *Cancer Cell*.

[120] Blum G, Von Degenfeld G, Merchant MJ, Blau HM, Bogyo M (2007). Noninvasive optical imaging of cysteine protease activity using fluorescently quenched activity-based probes. *Nature Chemical Biology*.

[121] Yee MC, Fas SC, Stohlmeyer MM, Wandless TJ, Cimprich KA (2005). A cell-permeable, activity-based probe for protein and lipid kinases. *Journal of Biological Chemistry*.

[122] Patricelli MP, Szardenings AK, Liyanage M (2007). Functional interrogation of the kinome using nucleotide acyl phosphates. *Biochemistry*.

[123] Cohen MS, Hadjivassiliou H, Taunton J (2007). A clickable inhibitor reveals context-dependent autoactivation of p90 RSK. *Nature Chemical Biology*.

[124] Kumar S, Zhou B, Liang F, Wang WQ, Huang Z, Zhang ZY (2004). Activity-based probes for protein tyrosine phosphatases. *Proceedings of the National Academy of Sciences of the United States of America*.

[125] Hekmat O (2005). Active-site peptide “fingerprinting” of glycosidases in complex mixtures by mass spectrometry. Discovery of a novel retaining beta-1,4-glycanase in Cellulomonas fimi. *Journal of Biological Chemistry*.

[126] Adam GC, Cravatt BF, Sorensen EJ (2001). Profiling the specific reactivity of the proteome with non-directed activity-based probes. *Chemistry and Biology*.

[127] Barglow KT, Cravatt BF (2004). Discovering disease-associated enzymes by proteome reactivity profiling. *Chemistry and Biology*.

[128] Barglow KT, Cravatt BF (2007). Activity-based protein profiling for the functional annotation of enzymes. *Nature Methods*.

[129] Speers AE, Adam GC, Cravatt BF (2003). Activity-based protein profiling in vivo using a copper(I)-catalyzed azide-alkyne [3 + 2] cycloaddition. *Journal of the American Chemical Society*.

[130] Speers AE, Cravatt BF (2004). Profiling enzyme activities in vivo using click chemistry methods. *Chemistry and Biology*.

[131] Rolén U, Kobzeva V, Gasparjan N (2006). Estrogen receptor co-activator (AIB1) protein expression by automated quantitative analysis (AQUA) in a breast cancer tissue microarray and association with patient outcome. *Mol Carcinog*.

[132] Jessani N, Humphrey M, McDonald WH (2004). Carcinoma and stromal enzyme activity profiles associated with breast tumor growth in vivo. *Proceedings of the National Academy of Sciences of the United States of America*.

[133] Sieber SA, Niessen S, Hoover HS, Cravatt BF (2006). Proteomic profiling of metalloprotease activities with cocktails of active-site probes. *Nature Chemical Biology*.

[134] Unwin RD, Evans CA, Whetton AD (2006). Relative quantification in proteomics: new approaches for biochemistry. *Trends in Biochemical Sciences*.

[135] Weerapana E, Speers AE, Cravatt BF (2007). Tandem orthogonal proteolysis-activity-based protein profiling (TOP-ABPP)—a general method for mapping sites of probe modification in proteomes. *Nature Protocols*.

[136] Jessani N, Niessen S, Wei BQ (2005). A streamlined platform for high-content functional proteomics of primary human specimens. *Nature Methods*.

[137] Nomura DK, Durkin KA, Chiang KP, Quistad GB, Cravatt BF, Casida JE (2006). Serine hydrolase KIAA1363: toxicological and structural features with emphasis on organophosphate interactions. *Chemical Research in Toxicology*.

[138] Zhang J, Valianou M, Cheng JD (2010). Identification and characterization of the promoter of fibroblast activation protein. *Frontiers in Bioscience*.

[139] Arai H (2002). Platelet-activating factor acetylhydrolase. *Prostaglandins and Other Lipid Mediators*.

[140] Tibes R (2006). Reverse phase protein array: validation of a novel proteomic technology and utility for analysis of primary leukemia specimens and hematopoietic stem cells. *Molecular Cancer Therapeutics*.

[141] Grote T, Siwak DR, Fritsche HA (2008). Validation of reverse phase protein array for practical screening of potential biomarkers in serum and plasma: accurate detection of CA19-9 levels in pancreatic cancer. *Proteomics*.

[142] Zhang L, Wei Q, Mao L, Liu W, Mills GB, Coombes K (2009). Serial dilution curve: a new method for analysis of reverse phase protein array data. *Bioinformatics*.

[143] Hennessy BT, Lu Y, Poradosu E (2007). Pharmacodynamic markers of perifosine efficacy. *Clinical Cancer Research*.

[144] Gonzalez-Angulo AM, Hennessy BT, Mills GB (2010). Future of personalized medicine in oncology: a systems biology approach. *Journal of Clinical Oncology*.

[145] Ding Z (2010). Physical association of PDK1 with AKT1 is sufficient for pathway activation independent of membrane localization and phosphatidylinositol 3 kinase. *PLoS ONE*.

[146] Carey MS (2010). Functional proteomic analysis of advanced serous ovarian cancer using reverse phase protein array: TGF-beta pathway signaling indicates response to primary chemotherapy. *Clinical Cancer Research*.

[147] Hennessy BT, Gonzalez-Angulo AM, Stemke-Hale K (2009). Characterization of a naturally occurring breast cancer subset enriched in epithelial-to-mesenchymal transition and stem cell characteristics. *Cancer Research*.

[148] Gonzalez-Angulo AM (2009). Androgen receptor levels and association with PIK3CA mutations and prognosis in breast cancer. *Clinical Cancer Research*.

[149] Stemke-Hale K, Gonzalez-Angulo AM, Lluch A (2008). An integrative genomic and proteomic analysis of PIK3CA, PTEN, and AKT mutations in breast cancer. *Cancer Research*.

[150] Bartholomeusz C (2010). PEA-15 inhibits tumorigenesis in an MDA-MB-468 triple-negative breast cancer xenograft model through increased cytoplasmic localization of activated extracellular signal-regulated kinase. *Clinical Cancer Research*.

[151] Brennan DJ (2010). Antibody-based proteomics: fast-tracking molecular diagnostics in oncology. *Nature Reviews Cancer*.

[152] Paweletz CP (2001). Reverse phase protein microarrays which capture disease progression show activation of pro-survival pathways at the cancer invasion front. *Oncogene*.

[153] Wulfkuhle JD, Aquino JA, Calvert VS (2003). Signal pathway profiling of ovarian cancer from human tissue specimens using reverse-phase protein microarrays. *Proteomics*.

[154] Rudelius M, Pittaluga S, Nishizuka S (2006). Constitutive activation of Akt contributes to the pathogenesis and survival of mantle cell lymphoma. *Blood*.

[155] Sheehan KM, Calvert VS, Kay EW (2005). Use of reverse phase protein microarrays and reference standard development for molecular network analysis of metastatic ovarian carcinoma. *Molecular and Cellular Proteomics*.

[156] Leng SX, McElhaney JE, Walston JD, Xie D, Fedarko NS, Kuchel GA (2008). ELISA and multiplex technologies for cytokine measurement in inflammation and aging research. *Journals of Gerontology *.

[157] Gowan SM, Hardcastle A, Hallsworth AE (2007). Application of meso scale technology for the measurement of phosphoproteins in human tumor xenografts. *Assay and Drug Development Technologies*.

[158] Yang DT (2011). Use of tissue microarray and automated quantitative analysis for screening and validation of potential biomarkers in mantle cell lymphoma. *Applied Immunohistochemistry & Molecular Morphology*.

[159] Kononen J, Bubendorf L, Kallioniemi A (1998). Tissue microarrays for high-throughput molecular profiling of tumor specimens. *Nature Medicine*.

[160] Brennan DJ, Kelly C, Rexhepaj E, Dervan PA, Duffy MJ, Gallagher WM (2007). Contribution of DNA and tissue microarray technology to the identification and validation of biomarkers and personalised medicine in breast cancer. *Cancer Genomics and Proteomics*.

[161] Camp RL, Neumeister V, Rimm DL (2008). A decade of tissue microarrays: progress in the discovery and validation of cancer biomarkers. *Journal of Clinical Oncology*.

[162] Choudhury KR, Yagle KJ, Swanson PE, Krohn KA, Rajendran JG (2010). A robust automated measure of average antibody staining in immunohistochemistry images. *Journal of Histochemistry and Cytochemistry*.

[163] Pages F, Berger A, Camus M (2005). Effector memory T cells, early metastasis, and survival in colorectal cancer. *New England Journal of Medicine*.

[164] Stromberg S, Bjorklund MG, Asplund C (2007). A high-throughput strategy for protein profiling in cell microarrays using automated image analysis. *Proteomics*.

[165] Brennan DJ, Ek S, Doyle E (2009). The transcription factor Sox11 is a prognostic factor for improved recurrence-free survival in epithelial ovarian cancer. *European Journal of Cancer*.

[166] Brennan DJ, Brandstedt J, Rexhepaj E (2010). Tumour-specific HMG-CoAR is an independent predictor of recurrence free survival in epithelial ovarian cancer. *BMC Cancer*.

[167] Camp RL, Chung GG, Rimm DL (2002). Automated subcellular localization and quantification of protein expression in tissue microarrays. *Nature Medicine*.

[168] McCabe A, Dolled-Filhart M, Camp RL, Rimm DL (2005). Automated quantitative analysis (AQUA) of in situ protein expression, antibody concentration, and prognosis. *Journal of the National Cancer Institute*.

[169] Dolled-Filhart M, Rydén L, Cregger M (2006). Classification of breast cancer using genetic algorithms and tissue microarrays. *Clinical Cancer Research*.

[170] Giltnane JM, Rydén L, Cregger M, Bendahl PO, Jirström K, Rimm DL (2007). Quantitative measurement of epidermal growth factor receptor is a negative predictive factor for tamoxifen response in hormone receptor-positive premenopausal breast cancer. *Journal of Clinical Oncology*.

[171] Gould Rothberg BE, Berger AJ, Molinaro AM (2009). Melanoma prognostic model using tissue microarrays and genetic algorithms. *Journal of Clinical Oncology*.

[172] Aitken SJ (2010). Quantitative analysis of changes in ER, PR and HER2 expression in primary breast cancer and paired nodal metastases. *Annals of Oncology*.

[173] Harigopal M, Heymann J, Ghosh S, Anagnostou V, Camp RL, Rimm DL (2009). Estrogen receptor co-activator (AIB1) protein expression by automated quantitative analysis (AQUA) in a breast cancer tissue microarray and association with patient outcome. *Breast Cancer Research and Treatment*.

